# How to report parameters and procedures for shockwave therapy in musculoskeletal disorders: A narrative review

**DOI:** 10.1097/MD.0000000000029664

**Published:** 2022-08-12

**Authors:** Athilas Braga de Menezes, Cláudio Gregório Nuerberg Back, Patricia Driusso, Richard Eloin Liebano

**Affiliations:** a Physiotherapeutic Resources Laboratory, Department of Physical Therapy, Federal University of Sao Carlos (UFSCar), Brazil; b Women’s health Research Laboratory, Department of Physical Therapy, Federal University of São Carlos (UFSCar), Brazil.

**Keywords:** musculoskeletal disorders, report parameters, shockwave therapy

## Abstract

Shockwave therapy (SWT) has been successful in the management of musculoskeletal conditions. The limitations of the use of SWT in clinical practice regard a lack of familiarity with the device and the lack of uniformity in information reported in scientific publications. Standardization in the reporting of these parameters could facilitate the reproduction and interpretation of data in future studies. Most studies fail to offer a detailed description of the parameters. Therefore, the aim of the present paper is to prepare a report on how to standardize the presentation of this information and serve a reference guide to report physical parameters and procedures of SWT when used on patients with musculoskeletal disorders. The terms were selected from the Medical Subject Headings database of controlled vocabulary. An extensive process of systematic searching of databases was performed, after which experts met and discussed on the main findings, and a consensus was achieved. SWT parameters were described, including the physiological meaning and clinical relevance of each parameter. Also, the description of patient and equipment positioning was added. The consensus-based guideline on how to report SWT parameters for the treatment of musculoskeletal conditions was developed to help clinicians and researchers.

## 1. Introduction

Shockwave therapy (SWT) has been successfully used for more than 20 years in the management of musculoskeletal conditions.^[1–3]^ SWT is a safe, noninvasive treatment option^[4,5]^ that is well accepted by patients for tendinopathy,^[6,7]^ myofascial pain,^[8–10]^ joint injuries^[11–13]^ and fractures with delayed union.^[14,15]^

The limitations of the use of SWT in clinical practice regard a lack of familiarity with the device and the lack of uniformity in information reported in scientific publications.^[4]^ Differences are found among studies available in the literature with regards to study design, protocol, application technique, duration of treatment and the parameters of the device. This heterogeneity makes it difficult for researchers and therapists to reproduce the methods described in articles and adopt a more assertive approach in clinical practice.^[16]^

Standardization in the reporting of these parameters could facilitate the reproduction and interpretation of data in future studies. Most studies fail to offer a detailed description of the energy, frequency and number of pulses, type and area of the tip, positioning of the patient, application site, type of device, type of applicator, inclusion of treatments combined with SWT, etc.^[16]^

This type of report has been published and widely used for other electrophysical agents, such as low-level laser therapy^[17]^ and electrotherapy for pelvic floor dysfunction.^[18]^ Therefore, the aim of the present paper is to prepare a report on how to standardize the presentation of this information and serve a reference guide for scholars, clinicians and researchers to report physical parameters and procedures of SWT when used on patients with musculoskeletal disorders.

## 2. Methods

The present literature review was conducted to identify published studies on shockwave therapy applied to musculoskeletal disorders and detect the type of information missing from these studies. Thus, the authors formed a workgroup to draft guidelines so that future studies can have more complete descriptions, enabling better reproduction of studies in both clinical practice and research. The members of the workgroup had expertise in fields related to musculoskeletal disorders (PD and CGNB) and SWT (REL and ABM) and participated in 4 meetings, at which the conception of the present report was planned. During the meetings, all authors contributed terms and expressions normally used in the literature of their fields. The terms were then grouped according to the order in which the items should be reported, as follows: frequency parameters, energy or energy density parameters, number of pulses, positioning of patient, application site, use of local anesthesia, coupling mechanisms, device, type of applicator, form of application, tip size, tip material, duration of application time and concomitant treatment. The process of discussing and writing the document followed a 3-step modified Delphi method and took place in May and June 2021.^[18–21]^

The first step was a literature review, conducted January to May 2021, in the following databases: Pubmed, PEDro, Web of Science and Embase. For all topics, we searched for existing systematic reviews and primary studies. We also consulted guidelines from international associations such as the World Confederation of Physiotherapy (WCPT), International Society for Medical Shockwave Treatment (ISMST) and Brazilian Medical Society for Shockwave Treatment (SBTOC). In addition, several. Textbooks that addressed electrophysical agents were also consulted.^[22–26]^ Terms related to the reporting of SWT for musculoskeletal disorders were selected from the controlled vocabulary of Medical Subject Headings (MeSH). Each electronic databank was searched with no restrictions imposed regarding language or year of publication. Definitions of established terms related to musculoskeletal disorders and SWT were used, when available; otherwise, the modified Delphi method was used for consensus.

The terms and definitions were then evaluated by specialists (two in musculoskeletal disorders and 2 in SWT). Two independent reviewers performed the selection process of primary studies by reviewing titles, abstracts and reading full texts, based on the proposed inclusion criteria described above. Reference lists of included studies were independently selected to identify possible studies not retrieved by the electronic search. In cases of a divergence of opinion, a physiotherapist unfamiliar with the terms of the field was consulted to verify the understanding of the term in question.

The terms used in the search strategy in the Pubmed, Web of Science and Embase databases were: (((Musculoskeletal disorder) OR (Musculoskeletal disorders) OR (Musculoskeletal system) OR (Musculoskeletal Pain) OR (Musculoskeletal disease) OR (Musculoskeletal diseases) OR (Musculoskeletal complaint) OR (Cumulative trauma disorders) OR (Skeletal muscle) OR (Orthopedic Disorders) OR (Orthopedic Disorder)) AND ((Randomized controlled trial) OR (Controlled clinical trial) OR (Comparative study) OR (Clinical trial) OR (Randomized) OR (Randomly) OR (Trial) OR (Groups)) AND ((Extracorporeal Shockwave Therapies) OR (Shockwave Therapies) OR (Shockwave Therapy) OR (Extracorporeal Shockwave) OR (Shock Wave Therapy) OR (Shock Wave Therapies) OR (Extracorporeal Shock Wave Therapy) OR (SWT) OR (ESWT) OR (rESWT) OR (Radial extracorporeal shock wave therapy) OR (Radial extracorporeal shock wave therapies) OR (Focused shock wave therapy) OR (Focused shock wave therapies)))

The second step was performed by a group of experts that discussed issues related to shockwave therapy in musculoskeletal disorders, while another group read the identified manuscripts and guidelines related to the topic. The first group consisted of 2 physical therapists (ABM and CGNB). Both professionals have more than 5 years of experience working in the area as clinicians and researchers. In addition, 2 physiotherapists specializing in musculoskeletal disorders (REL and PD) with extensive experience in the field complemented the initial draft with additional modifications. At this stage, the experts held several online meetings to discuss the findings and vote on the recommendations. All terms that were approved by the majority of experts were considered.

The third step was a meeting was held to revise the document and propose final recommendations. This step consisted of gathering information collected during the review of the literature. The authors opted for 2 tables—one focused on the parameters of SWT and another describing all that should be reported/performed for the administration of SWT in musculoskeletal disorders.

## 3. Results

Table 1 displays the definitions of the parameters for SWT and the clinical relevance of each parameter. Table 2 displays the results of how the use of SWT and its parameters should be described in studies on musculoskeletal disorders.

**Table 1:** Definitions and clinical relevance of each parameter of shockwave therapy equipment.

Parameter (unit of measurement)	Description	Clinical relevance
Frequency (Hz)	Number of pulses per second^[22,23]^	Frequency affects the penetration capacity of the energy into biological tissue, with a lower frequency achieving greater energy penetration.^[27]^
Acoustic energy or acoustic intensity	Energy transmitted per unit of area per pulse.	The therapeutic effects of the shockwaves depend on the energy distributed in a broad or focused area of the treatment zone.^[23]^
May be expressed as: energy density (mJ/mm²) or pressure (Bar) or total energy (mJ)	Future studies need to add an energy equivalence table for the device employed (pressure in bar × energy density in mJ/mm² × energy in mJ) to enhance the external validity of the study.	With better delivery of energy density to the tissue, the significant tissue effect generated is the mechanical effect and the resulting cavitation of the negative phase of the propagation of the shockwave, which can have important consequences regarding the therapeutic bioeffect.^[28–30]^
	Total energy is defined as energy multiplied by the number of pulses.^[4,28]^	
Shockwave generator	Focused (Focal) radial^[22]^	The shockwave generator determines the depth and concentration of energy of the wave. A focused shockwave generator tends to reach deeper tissues and concentrate the energy, meaning that the mechanical effect and cavitation occur at a farther distance from the applicator. A radial shockwave generator tends to reach more surface tissues with less concentration of energy; thus, the mechanical effect and cavitation occur closer to the applicator.^[29,30]^
		Radial shockwaves tend to have a larger, more dispersed area for the distribution of cavitation—or divergence. With a focal shockwave focal, there is more convergence at the site that will have the cavitation, with a smaller area of energy concentration, requiring considerable application precision.^[22]^ This precision is extremely important to avoid/prevent possible tissue damage due to the high concentration of energy in a small area and also so that the energy reaches the target tissue.
Pulses/ Shots	Number of pulses during treatment	It is through the pulse that the mechanical energy is transferred to the tissue. Thus, the acoustic intensity, number of pulses and form of application determine the amount of energy transferred to the tissue. After the transference of energy, each tissue may respond in a different manner, depending on the focus of the treatment—whether to accelerate the tissue regeneration process or achieve the disintegration of calcific conditions.^[1,2]^

**Table 2:** Description of each application parameter that needs to be reported in studies employing shockwave therapy.

Item	Parameter	Description
Patient	Positioning: describe the position in which the patient received shockwave therapy	Prone or supine, sitting or standing
	Skin preparation: describe how the skin was prepared to receive the applicator	Use of support to make patient comfortable
		Furniture/device on which the patient is positioned (chair or examining table)
Localization of application site	Use of ultrasound or manual palpation to identify the target structure	Detect the desired structure for application.
Local anesthesia	Report the use of local anesthesia.	If used, describe the anesthetic and quantity; Describe patient comfort (whether patient felt pain and degree of pain)
Coupling mechanism	Use of conductive gel, lotion or coupling bags	Site of use of gel or bags, brand, duration of use
Device	Complete description of device	Commercial name, brand and model
		Country of manufacturer
		Periodic calibration (if performed)
Type of applicator	Electrohydraulic (focal)	The way that the energy is generated varies depending on the type of applicator. Thus, clinicians should describe what applicator was used for the administration of SWT.
	Electromagnetic (focal)	
	Piezoelectric (focal)	
	Pneumatic (radial)	
	Electromagnetic (radial)^[25]^	
Form of application	Static	Depends directly on the area and objective of therapy.
	Dynamic	
Application area	Describe the area of application of SWT	Depends directly on the form of application and number of pulses in a predetermined area.
		Ex.: 2000 static shots in the gluteal region is different from 2000 scanning shots in the same region.
Tip size and shape	Describe the size of the tip in mm² and its shape (convex, flat or concave)	The stimulus on the tissue and patient comfort can be affected depending on the size of the tip.
		The concentration of energy can be affected by the size of the tip. Smaller tips are used for a greater concentration of energy, but this has a direct impact on the patient’s sensory level.
		The shape of the tip directly affects the depth and concentration of energy. Tip shape alters the form of energy transference to the target tissue.
Tip material	Clarify the material of the tip used (metal or polyacetal)	The depth of the wave can vary depending on the tip material, type of tissue and adjusted energy.
Treatment	Duration of application	Time in minutes
	Duration of therapy	Number of sessions in which shockwave therapy was administered
	Interval between sessions	Number of hours or days between sessions
	Report of patient discomfort	Assessment of discomfort during treatment
		Instrument used to assess discomfort (self-report, visual analog scale, etc)
	Reported/observed side effects	Any side effects reported or observed during treatment should be described: considerable discomfort after the end of the treatment session, petechiae, redness, etc)
	Patient adherence to treatment	Percentage of patients who adhered to the entire treatment
		Number of effective sessions/number of sessions planned
	Combined treatment: describe any type of therapy performed simultaneously to shockwave therapy	Home-based and/or in-person exercises, medications, educational sessions or any other type of therapy used; Describe each therapy in complete detail.
	Result/outcome: describe what variable is the primary outcome and the methods used to assess the outcome.	For scientific studies: Functioning, quality of life, pain at rest and when performing activities, range of motion, muscle strength, calcific changes, adverse events, etc.
		For clinical practice: Clearly indicate the main objective of therapy.

## 4. Discussion

Therapists and researchers should always consider the indications and contraindications of shockwave therapy (SWT) to ensure that the patient is eligible for treatment.^[22–26]^ Researchers and clinicians should certify that all exclusion criteria to the use of SWT were evaluated, such as the use of a pacemaker or coagulation disorders.^[24,26]^ If SWT is applied directly to the skin, it is necessary to assess contraindications, such as uncorrected bleeding disorders, severe peripheral vascular disease or acute infections.^[2,3,28]^ After this initial evaluation, the clinician/researcher must obtain consent from the patient prior to initiating the intervention. An adequate, precise assessment of the entire region to be treated is mandatory to determining the most appropriate type of treatment or study/clinical trial design. The application site^[31,32]^ and positioning of the patient^[33,34]^ must also be taken into account. This type of evaluation should be performed by a specially trained health care provider (e.g., physiotherapist, nurse, physician, etc) to ensure a consistent, precise intervention.

Recommendations regarding the safe handling of the device should be followed to minimize the risk of injury to the patient.^[23,25,26]^ Each country has specific guidelines for maintenance and inspection that must be respected, which is why it is important to report the type of device that was used. In most countries, necessary maintenance depends on the number of pulses that the apparatus can withstand to avoid device failures. Operating manuals, models, serial numbers and inspection certificates need to be up to date.

The therapeutic effects of SWT and side effects in different types of tissues have not been fully clarified.^[22,35,36]^ It is hypothesized that the therapeutic effects of shockwaves may be due to the direct effect caused by the mechanical pressure and tension that the waves exert on the tissue, with a change in acoustic impedances, and an indirect effect due to the formation of cavitation bubbles that induce shearing forces at the site upon bursting.^[37,38]^

Numerous factors can affect the success or failure of SWT, such as the use of local anesthesia, the form of application, tip size and tip material. Some studies have demonstrated that the effect of SWT is dose dependent and can activate or sensitize nociceptive fibers. The use of an anesthetic can substantially alter these biological responses to SWT.^[39–41]^ Some clinicians/researchers use anesthetics when employing focused applicators, since the application is deeper and, at times, painful or uncomfortable. The use of anesthetics in such cases is to prevent probable side effects during and after administration, such as intense pain at rest or when moving the treated site, and facilitate patient adherence to treatment.

To date, no clinical studies have evaluated whether tip size and material and form of application exert a direct or indirect influence on the therapeutic and physiological effects. Tip size affects local energy density. Thus, smaller tips are used to obtain a greater concentration of energy, which has a direct impact on the patient’s sensory level. The depth of the wave can vary with the tip material, which can also exert a direct influence on patient comfort during the administration of SWT. Regarding the form of application, the 2 possibilities are static and dynamic (scanning over large area horizontally and vertically). We believe that the form of application can exert a direct influence on energy delivery to the target tissue, as scanning can lead to a considerable variation in the distribution of energy delivered to the tissue.

The coupling mechanisms of the devices for the emission of the shockwaves is another factor that needs to be reported. Formerly, SWT was applied in water immersion baths, especially devices developed for lithotripsy. Today, however, the technology does not require a large device and SWT involves the application of a conductor gel with radial devices or coupling bags with focused devices. However, most studies fail to describe this aspect, which can confuse clinicians who have no knowledge on the device and the best form of application.^[22,23]^

Current guidelines are ambiguous regarding the ideal number of sessions, quantity of pulses per session, duration of the application time and interval between sessions.^[23,42–44]^ The lack of consensus on these aspects has generated considerable divergences in the literature, which affects clinical decision making. Thus, a full description of these parameters should always be performed to enable the reproducibility of the application.

Among all these topics, 2 warrant greater attention: the description of the frequency^[5,45]^ and the failure to provide an energy equivalence table of the device used.^[5,46]^ Frequency is directly linked to the velocity of energy delivered to the tissue without affecting the dose offered. Thus, the target tissue is exposed to energy for a shorter time when high frequencies are used compared to low frequencies.^[45]^ Hence, a higher frequency translates to a shorter application time with the same number of pulses.

The report of the dosimetry is found in a large part of the studies and expressed as energy (mJ), energy density (mJ/mm²) or pressure (bar). This depends on the device being used and type of applicator. However, one should bear in mind that the device available to the clinician/researcher may not have the same configurations, which hinders reproducibility. This can be minimized in future studies by added an energy equivalence table, which would enable greater reproduction of the findings of a clinical study and enhance its external validity.

## 5. Conclusions

In the present article, we created tables for the successful reporting of parameters and procedures of shockwave therapy for musculoskeletal disorders. We recommend the recognition of these standards in publications related to the use of SWT. We hope that the present report can assist researchers and clinicians involved in the rehabilitation of patients with musculoskeletal disorders, enabling the adequate, complete description of parameters to ensure the reproducibility of the methods, a critical analysis of the results and the advancement of knowledge in this field.

## Author contributions

Athilas Braga de Menezes - Investigation; Methodology

Claudio Gregório Nuerberg Back - Formal analysis, Resources

Patricia Driusso - Conceptualization

Richard Eloin Liebano - supervision, Project administration

All authors - Writing, review & editing
